# Supplementary material for: Amide-Containing Bottlebrushes via Continuous-Flow Photoiniferter Reversible Addition–Fragmentation Chain Transfer Polymerization: Micellization Behavior
Source: Polymers (Basel). 2023 Dec 31;16(1):134. doi: 10.3390/polym16010134 (PMC10780833; doi:10.3390/polym16010134)
Supplement: Supplementary file 1 [file polymers-16-00134-s001.zip › polymers-2782598-supplementary.pdf]

Supplementary Information for:

Amide-containing bottlebrushes via continuous flow PI-RAFT polymerization: micellization behavior

Alexey Sivokhin [1,*], Dmitry Orekhov [1], Oleg Kazantsev [1], Ksenia Otopkova [1], Olga Sivokhina[2], Ilya Chuzhaykin [1], Alexey Ovchinnikov [1], Olga Zamyshlyaeva[3], Irina Pavlova [1], Olga Ozhogina [1], Maria Chubenko [1]

[1] Research Laboratory "New Polymeric Materials", Nizhny Novgorod State Technical University n.a. R.E. Alekseev, 24 Minin Street, 603155 Nizhny Novgorod, Russia
[2] V.A. Kargin Research Institute of Chemistry and Technology of Polymers with Pilot Plant, 606000 Dzerzhinsk, Nizhegorodskaya obl., Russia
[3] Department of High Molecular Compounds and Colloidal Chemistry, Faculty of Chemistry, Lobachevsky State University, Gagarina pr. 23, Nizhny Novgorod 603950, Russia
*Correspondence: sivokhin@dpingtu.ru; Tel.: +7-831-334-7166

Characterization techniques

The concentrations of the monomers in reaction mixtures were measured by HPLC using a Shimadzu Prominence chromatographic system equipped with a refractometer and matrix UV detector, a thermostat and a Kromasil 100-5-C18 4.6 x 250 mm column. Acetonitrile was used as an eluent, the flow rate was 0.9 ml/min, and the thermostat temperature was 55°C.

The compositions of copolymers at each conversion were calculated from the monomer consumption. At certain intervals, the current monomer concentrations were determined by HPLC; the copolymer composition (m_1) and the current conversion were calculated according to the following equations:

$$m_1 = \frac{M_1^0 - M_1}{(M_1^0 - M_1) + (M_2^0 - M_2)} \cdot 100\%,$$

$$Conversion = \frac{(M_1^0 - M_1) + (M_2^0 - M_2)}{M_1^0 + M_2^0} \cdot 100\%,$$

where m_1 is the content of monomer 1 units in a copolymer, mol%; M_1^0 and M_2^0 are the initial concentrations of monomers 1 and 2 in the feed, mmol/g; M_1 and M_2 are the current concentrations of monomers 1 and 2, mmol/g.

All copolymers were characterized by ^{1}H NMR spectroscopy (Figures S1-S7). For P6 and P7, the copolymer compositions were determined based on the ratio of the signal intensities of the protons corresponding to the methyl group (e) contained in MPEGMA units, methylene group (c) contained in both MPEGMA and AOEGMA units and methyl group (k) contained in N-methylacrylamide units.

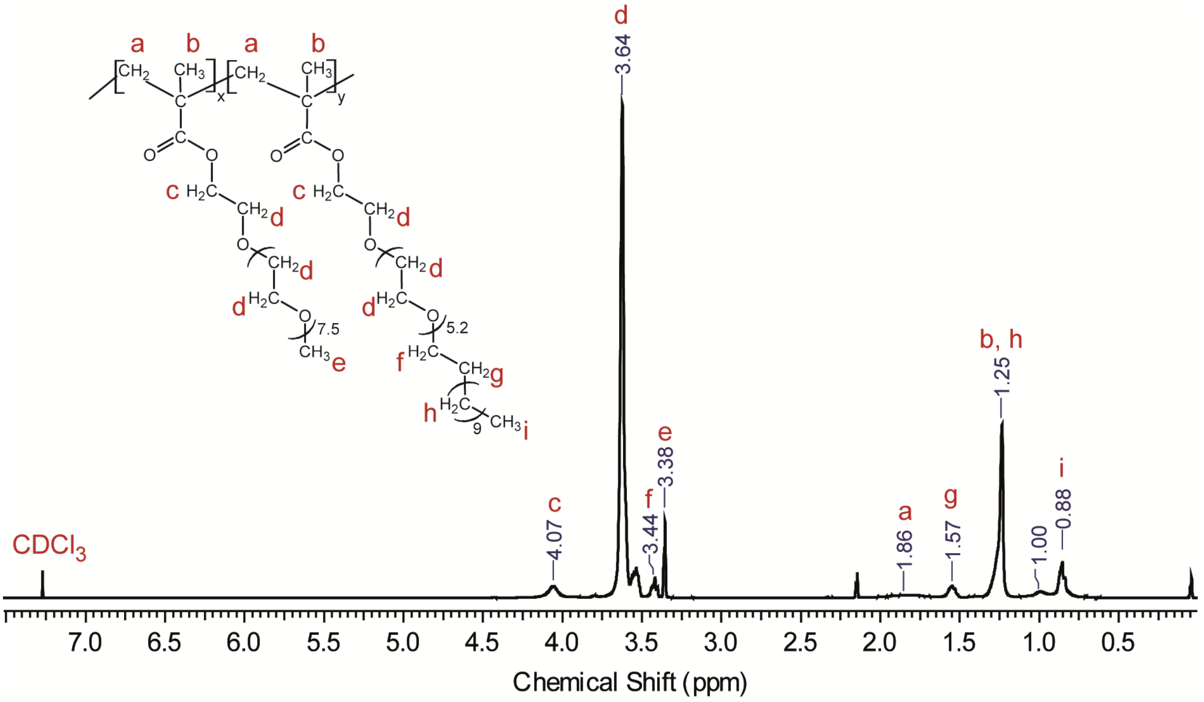

Figure S1. ^{1}H NMR spectra of polymer P1 in $CDCl_3$

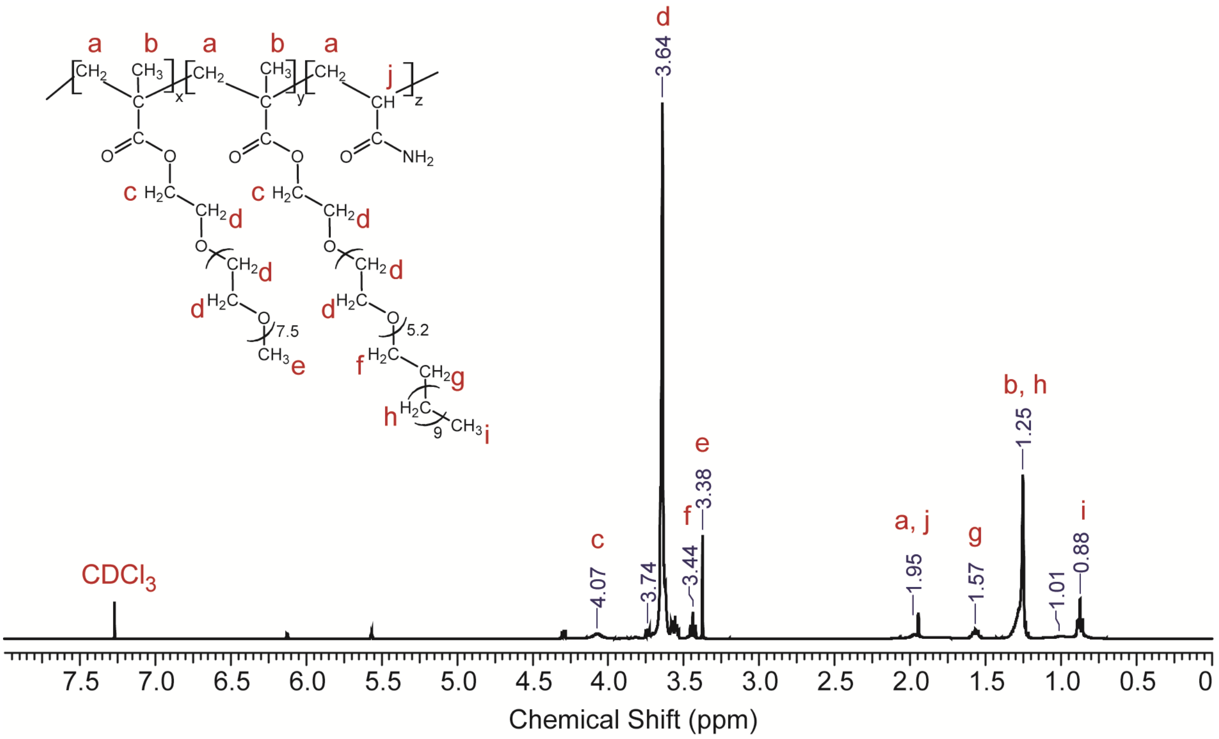

Figure S2. ^{1}H NMR spectra of polymer P2 in $CDCl_3$

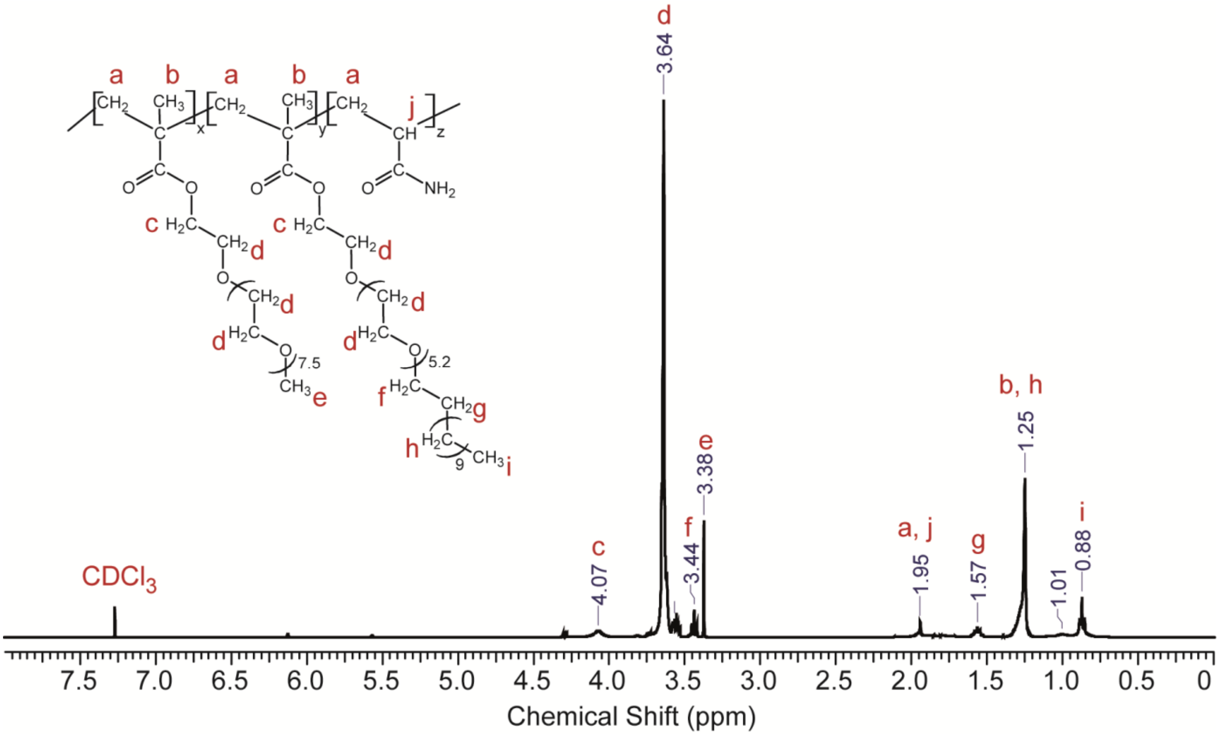

Figure S3. ^{1}H NMR spectra of polymer P3 in $CDCl_3$

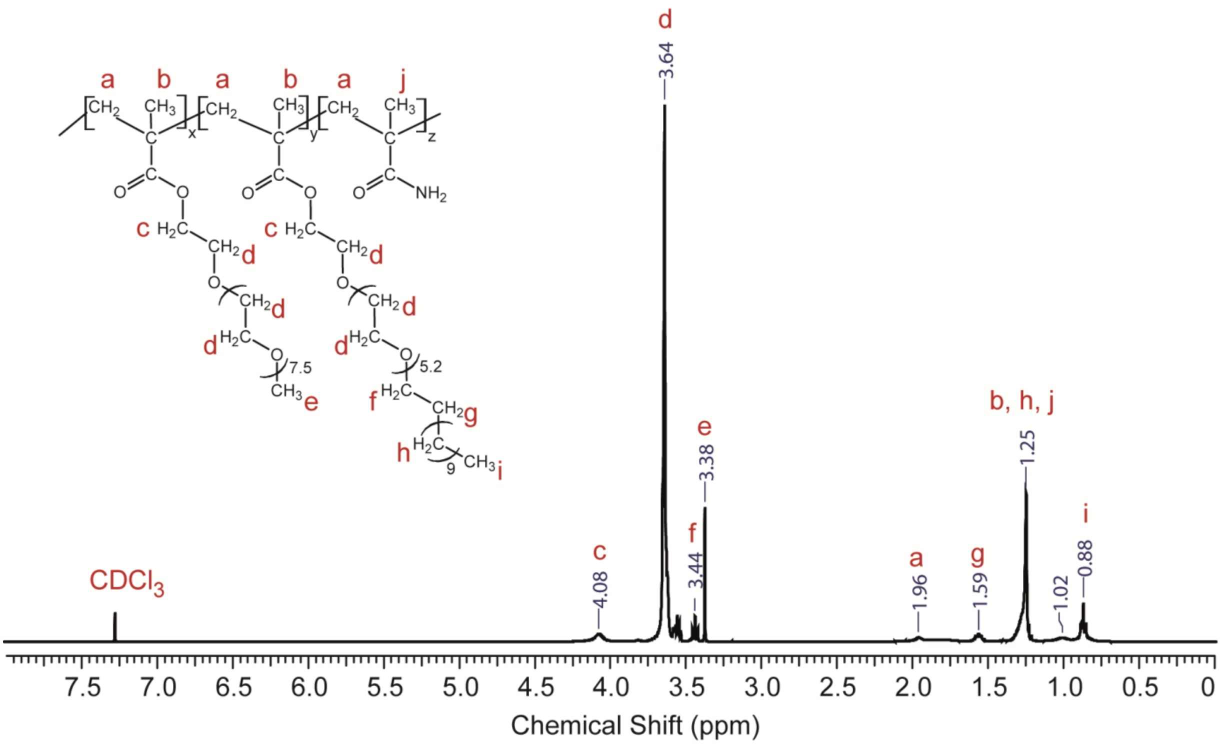

Figure S4. ^{1}H NMR spectra of polymer P4 in $CDCl_3$

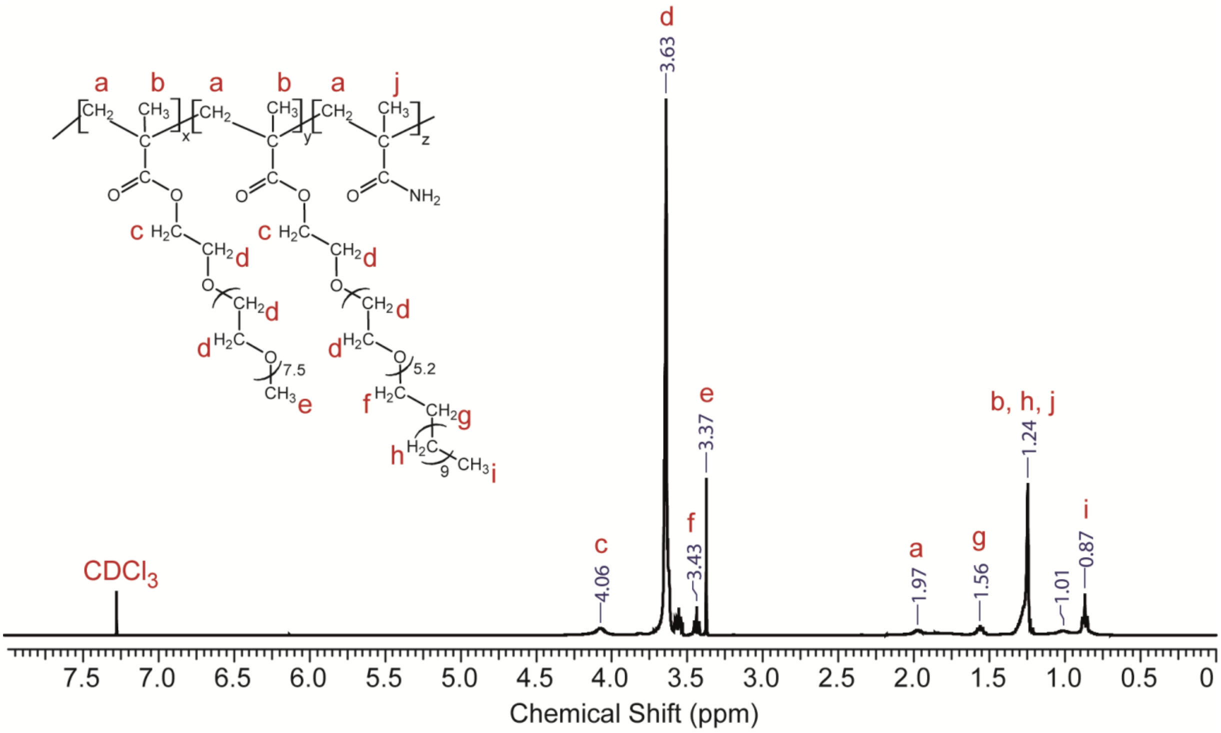

Figure S5. ^{1}H NMR spectra of polymer P5 in $CDCl_3$

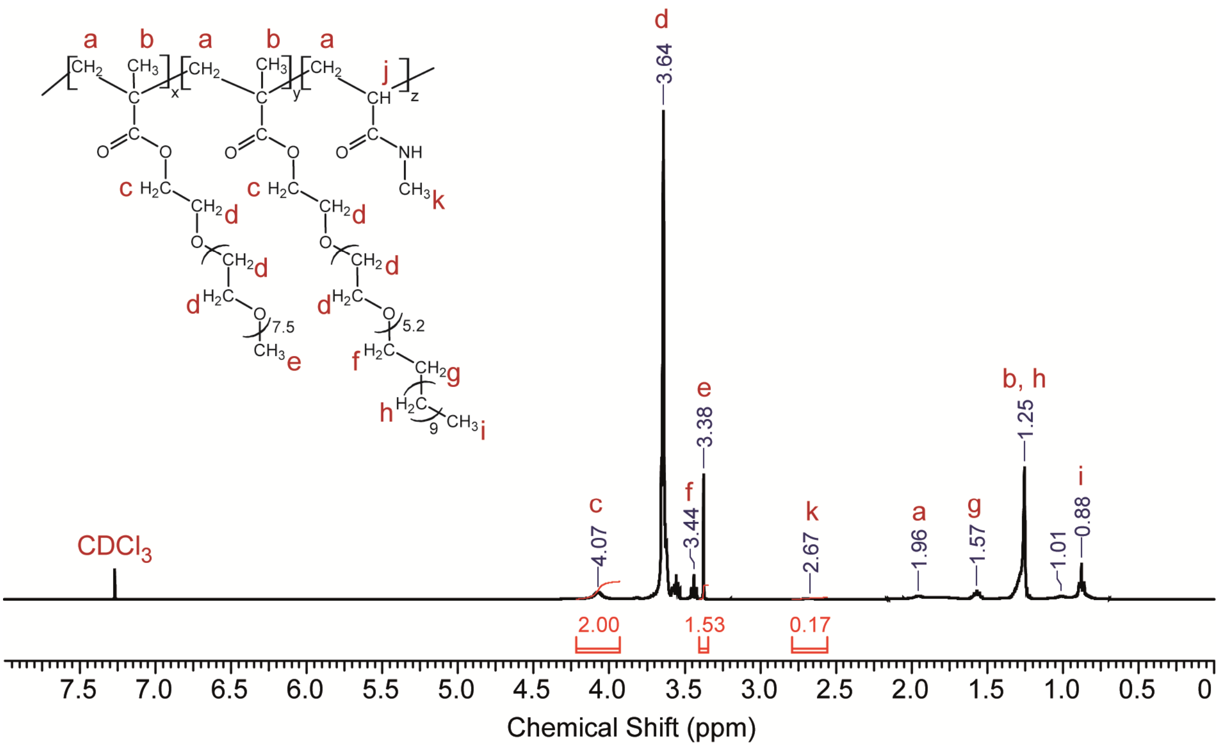

Figure S6. ^{1}H NMR spectra of polymer P6 in $CDCl_3$

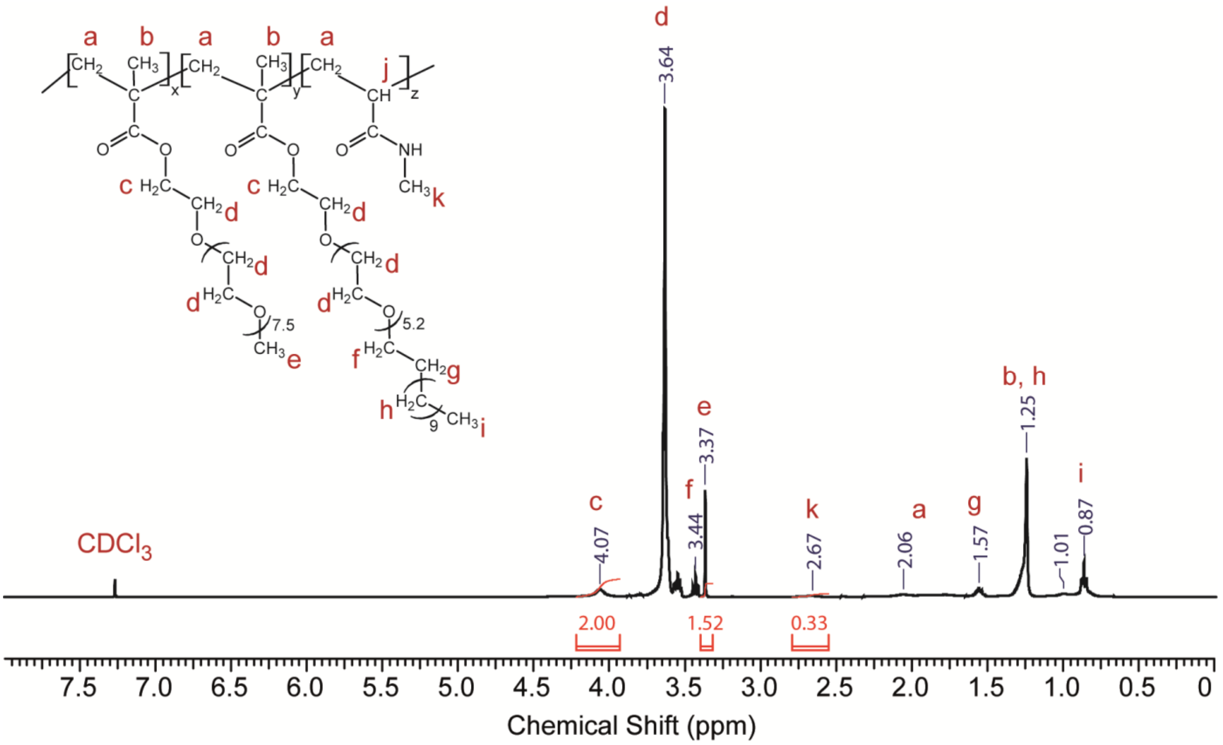

Figure S7. ^{1}H NMR spectra of polymer P7 in $CDCl_3$
